# Higher risk for acute childhood lymphoblastic leukaemia in Swedish population centres 1973-94

**DOI:** 10.1038/sj.bjc.6690007

**Published:** 1999-09-24

**Authors:** U Hjalmars, G Gustafsson, on behalf of the Swedish Child Leukaemia Group

**Affiliations:** Department of Pediatrics, Östersunds Hospital, Östersund, S-831 83, Sweden; Child Cancer Research Unit, Karolinska Hospital, Stockholm, S-171 76, Sweden

**Keywords:** epidemiology, childhood leukaemia, geographical information system, population density, infection

## Abstract

A population-based sample of acute childhood leukaemia cases in Sweden 1973–94 was analysed by a geographical information system (GIS) for spatial leukaemia distribution in relation to population density. The annual incidence rate for acute lymphoblastic leukaemia (ALL) was 3.6, and for acute non-lymphoblastic leukaemia (ANLL) 0.7, cases per 100 000 children. Incidence rates in population centres, constituting 1.3% of Sweden's land area and approximately 80% of the population, compared with the rest of Sweden showed a statistically significant excess of ALL [odds ratio (OR) 1.68; 95% confidence interval (CI) 1.44–1.95], but not ANLL (OR 1.13; 95% CI 0.98–1.32). An increasing trend, however not statistically significant, was found for ALL incidence with both increasing population density in parishes and increasing degree of urbanity in municipalities. These findings support the theories that some environmental factors associated with high population density, such as infectious agents, may be of aetiological importance for childhood acute lymphoblastic leukaemia. © 1999 Cancer Research Campaign


*British Journal of Cancer* (1999) **79**(1), 30–33
© 1999 Cancer Research Campaign

### Higher risk for acute childhood lymphoblastic leukaemia in Swedish population centres 1973–94

**U Hjalmars[1] and G Gustafsson[2] on behalf of the Swedish Child Leukaemia Group**

[1]Department of Pediatrics, Östersunds Hospital, S-831 83 Östersund, Sweden; [2]Child Cancer Research Unit, Karolinska Hospital, S-171 76 Stockholm, Sweden

**Summary** A population-based sample of acute childhood leukaemia cases in Sweden 1973–94 was analysed by a geographical information system (GIS) for spatial leukaemia distribution in relation to population density. The annual incidence rate for acute lymphoblastic leukaemia (ALL) was 3.6, and for acute non-lymphoblastic leukaemia (ANLL) 0.7, cases per 100 000 children. Incidence rates in population centres, constituting 1.3% of Sweden's land area and approximately 80% of the population, compared with the rest of Sweden showed a statistically significant excess of ALL [odds ratio (OR) 1.68; 95% confidence interval (CI) 1.44–1.95], but not ANLL (OR 1.13; 95% CI 0.98–1.32). An increasing trend, however not statistically significant, was found for ALL incidence with both increasing population density in parishes and increasing degree of urbanity in municipalities. These findings support the theories that some environmental factors associated with high population density, such as infectious agents, may be of aetiological importance for childhood acute lymphoblastic leukaemia.

**Keywords:** epidemiology; childhood leukaemia; geographical information system; population density; infection

Despite decades of intensive investigation, the aetiology of acute childhood leukaemia is still an enigma. It is the dominating childhood malignancy, accounting for about one-third of all childhood cancer in developed countries (Parkin et al, 1988; Ries et al, 1994). Many theories have been presented concerning the cause or promotion of leukaemia, but little is known for certain about the aetiological factors and mechanisms involved. Established causes represent a minority of all cases. A number of biological, physical and chemical agents have been suggested as risk factors, but the results of numerous investigations are in many cases unconvincing (Ross et al, 1994). Infectious involvement in the aetiology of leukaemia is an old hypothesis (Aubertin and Grellety, 1923) that has been revived during recent years and is supported by results from several studies (Greaves, 1986; Kinlen et al, 1990; Alexander, 1993; Greaves and Alexander, 1993; Kinlen, 1995). There is, however, as yet no direct biological evidence for an infectious aetiology. The results of investigations into a possible relation between the incidence of childhood leukaemia and high population density are contradictory (Bithell, 1990; Muirhead, 1995) Rapid population mixing, particularly in rural areas, has been associated with the increase in incidence of childhood acute lymphoblastic leukaemia (Kinlen et al, 1990; Kinlen, 1995,1997).

The main topic addressed in the present study was the possible relation between population density and incidence rates of ALL and ANLL.

### MATERIAL AND METHODS

### Material

The study was population based and comprised all 1593 cases of acute leukaemia in children aged 0–15 years diagnosed in Sweden between 1973 and 1994. The register is updated annually and is based on reports from all Swedish hospitals involved in the diagnosis and treatment of childhood leukaemia (Gustafsson and Wranne, 1993). Repeated cross-checking against the Swedish Cancer Registry verified the completeness of the study base. Different variables such as age, sex, date of diagnosis and haematological values were included. Information about county, municipality and parish at diagnosis were registered for all patients. For 99.5% of the patients, the place of residence at diagnosis with address and precise geographical coordinates were determined. Detailed information on the background population of 1.7 million children was available. Annual age-stratified data for counties, municipalities and parishes were used. All real estates in Sweden that were populated by children within the age group 0–15 years for 1978, 1982 and 1988 were pinpointed to precise geographical coordinates. The data used were obtained from Statistics Sweden and The Centre for Real Estate Board.

### Methods

Three different approaches were used to compare leukaemia risk for children in areas of varying population densities:

(1) The incidence in population centres was compared with incidence in the rest of Sweden. A population centre is defined by Statistics Sweden as a densely populated area with 200 inhabitants or more in which the distance between dwellings was not more than 200 m. There are about 2000 such population centres in Sweden representing approximately 80% of the total population, but only 1.3% of the land area.
(2) Comparisons were made between parishes with different population counts. Population densities in parishes were classified into three groups: <400, 400–<800 and ≥800 children per km². These classes were based on counts of the total childhood population under the age of 16 as a mean for the years 1976, 1982, 1988 and 1994.

*Received 27 January 1998*
*Revised 1 June 1998*
*Accepted 11 June 1998*

*Correspondance to:* U Hjalmars

(3) Leukaemia rates were compared between three different types of municipalities according to the classification codes of Statistics Sweden that define all Swedish municipalities as urban, semiurban or rural.

### Geographical information systems (GIS)

For the spatial analysis of population centres compared with non-centre areas, a geographical information system (GIS) was used, namely ARC/INFO. A GIS can be described as a computer-assisted information management system for georeferenced data including automated systems for the capture, storage, retrieval, analysis and display of spatial data. The range of analytical complexity for GIS is wide; from simple visualization to sophisticated statistical methods for analysis of spatial distribution and interaction. The main use of GIS is still as a hypothesis-generating instrument. In this study, we utilized the GIS to investigate a possible relation between population centres and childhood leukaemia by overlaying digital maps with geographical coordinates for all leukaemia cases and background population, pinpointed to real estates, with the borders of population centres.

### Statistical methods

Odds ratios were calculated when comparing leukaemia risk in population centres with the rest of Sweden, in parishes with varying population density and for the different municipality types (Kahn and Sempos, 1989).

A score test by logistic regression was used for the trend analyses for parishes and municipalities.

### RESULTS

There were 1593 cases of acute leukaemia diagnosed in Sweden in children below 16 years of age during the 22-year study period, 1973–94. Of these, 1346 cases (84%) were classified as acute lymphoblastic leukaemia (ALL) and 247 cases (16%) as acute non-lymphocytic leukaemia (ANLL). Children with Down's syndrome and a diagnosed congenital transient leukaemia were not included in the material (Weinstein, 1978). The overall annual incidence rate for ALL was 3.6 [95% confidence interval (CI) ± 0.2] and for ANLL 0.7 (95% CI ± 0.1) cases, per 100 000 children. Sex ratio (boys/girls) was statistically significant for boys in ALL: 1.2 [odds ratio (OR) 1.14; 95% CI 1.02–1.27], but not in ANLL: 0.9 (OR 0.8; 95% CI 0.64–1.05).

On comparing the risk of leukaemia in population centres with the risk in the rest of Sweden, a statistically significant difference was found for ALL (OR 1.68; 95% CI 1.44–1.95) but not for ANLL (OR 1.13; 95% CI 0.98–1.32) (Table 1). The increase was confined to all three age groups 0 to <6, 6 to <10 and 10 to <16 years. A comparison of ALL risk in parishes with different child population density (<400, 400–< 800 and > 800 per km$^2$) revealed an increased incidence associated with a higher population density, although not statistically significant (Table 2). A third, somewhat cruder, comparison using municipality codes (rural, semiurban and urban) also showed a statistically non-significant relation between the degree of urbanity and increased incidence of childhood ALL (Table 3). A score test for trends of ALL risk related to higher population density in parishes and higher degree of urbanity in municipalities showed an increase, with *P*-values of 0.46 and 0.17 respectively. The test used even scoring between the three categories. No consistent relation between ANLL and population density parameters was found.

Earlier analyses performed on the childhood leukaemia material in Sweden for more limited time periods have not shown statistically significant spatial geographical clusters of ALL or ANLL (Waller et al, 1995; Hjalmars et al, 1996). There are, thus, no indications that the higher risk is confined to specific population centres.

The question of a possible association of higher ALL risk with large increases in small area populations was considered. There has not been any unusual population mixing in Sweden during the period studied. The ALL risk was not statistically significantly increased for the 10% of parishes in Sweden that had the largest increase in child population during the study period (OR 1.16; 95% CI 0.97–1.37). The 10% of parishes with the highest incidence rates showed a 0.6% increase of child population compared with a 2.1% decrease in the rest of the country.

### DISCUSSION

Environmental factors are considered to be involved in leukaemia aetiology. Analyses of some agents have shown convincing evidence of aetiological importance. For the majority of factors, however, a causal link is not proven beyond doubt (Ross et al, 1994; Alexander, 1995). The sharp incidence peak for ALL at 2–3 years of age may indicate prenatal or early post-natal exposure to environmental aetiological factors. Much attention has in recent years been drawn to the theories of infectious aetiology. Kinlen et al (1990) have, in several studies, shown an increased risk of leukaemia among children in geographical areas experiencing intense population mixing. Thus, there is substantial evidence in favour of infection as an aetiological factor, but the exact nature of the contagious environmental hazards involved are as yet unidentified. No specific transmissible agent has been identified, and proof of the existence of infectious agents in this disease would not exclude the operation of other environmental factors. In studies by Kinlen et al (1990; Kinlen, 1995, 1997), extreme situations of population mixing have been analysed. Earlier results concerning relation between population density and leukaemia incidence have

**Table 1** Acute leukaemia incidence rates in densely populated areas compared with the rest of Sweden. Population counts, cases and incidence rates (cases per 100 000)

| | Population (mean 1978, –82 and –88) | ALL cases | ANLL cases | ALL incidence | 95% CI | ANLL incidence | 95% CI |
|---|---|---|---|---|---|---|---|
| Dense population | 1 302 858 | 1145 | 192 | 3.99 | ±0.23 | 0.67 | ±0.09 |
| Non-dense population | 377 996 | 198 | 49 | 2.38 | ±0.33 | 0.59 | ±0.16 |
| Total | 1 680 854 | 1343 | 241 | 3.63 | ±0.19 | 0.65 | ±0.08 |
| [Odds ratio (dense/non-dense)] | | | | 1.68 (1.44–1.95) | | 1.13 (0.98–1.32) | |

© Cancer Research Campaign 1999

**Table 2** Acute leukaemia in parishes with different population density. Population, cases and annual incidence rates (cases per 100 000)

| Population density (Age 0–15 per km²) | Population (mean 1976–94) | ALL cases | ANLL cases | ALL incidence | 95% CI | ANLL incidence | 95% CI |
|---|---|---|---|---|---|---|---|
| <400 | 1 562 072 | 1220 | 230 | 3.55 | ±0.20 | 0.67 | ±0.09 |
| 400–800 | 115 104 | 92 | 10 | 3.63 | ±0.74 | 0.39 | ±0.24 |
| > 800 | 37 791 | 34 | 7 | 4.09 | ±1.37 | 0.84 | ±0.62 |
| Total | 1 714 966 | 1346 | 247 | 3.57 | ±0.19 | 0.65 | ±0.08 |

**Table 3** Acute leukaemia in municipalities with different urbanicity. Population, cases and annual incidence rates (cases per 100 000)

| | Population | ALL cases | ANLL cases | ALL incidence | 95% CI | ANLL incidence | 95% CI |
|---|---|---|---|---|---|---|---|
| Rural | 298 075 | 218 | 45 | 3.32 | ±0.44 | 0.69 | ±0.20 |
| Semiurban | 218 470 | 164 | 41 | 3.41 | ±0.52 | 0.85 | ±0.26 |
| Urban | 1 199 216 | 964 | 161 | 3.65 | ±0.23 | 0.61 | ±0.09 |
| Total | 1 715 761 | 1346 | 247 | 3.57 | ±0.19 | 0.65 | ±0.08 |

been inconsistent. A study from three metropolitan regions of the USA showed a statistically significant increasing trend in incidence rates for ALL and non-Hodgkin's lymphoma with increasing population density (Muirhead, 1995). In Australia, a higher incidence of ALL was found in Brisbane City compared with the surrounding area (McWhirter & Bacon, 1980). In contrast to this, results from Britain show higher incidence rates of childhood leukaemia in rural compared with urban areas (Draper, 1991; Langford and Bentam, 1993). The overall incidence of childhood leukaemia in Sweden is comparatively high; it is considerably higher than in Britain and among the highest in the world (Parkin et al, 1988). The age-adjusted incidence rate for ALL for the age group 0–14 years was 3.3, and 0.6 for ANLL, in England and Wales (Stiller and Draper, 1998). The rates for the same age groups in Sweden were 3.8 and 0.7 respectively.

Corrections for socioeconomic or other possible confounding factors were not carried out in the present study because such factors were not registered. During the study period the socio-economic standard in Sweden was high and stable.

A statistically significant enhanced risk of ALL in population centres was found. The analyses were accurate by using precise coordinates for each case and all children in the background population. The results from the two other comparisons, using somewhat cruder methods of assessment of relationship between population density and leukaemia incidence, were concordant with this result, but were within possible random variation. These are more traditional methods for studying the relation between population density and disease occurrence. As the geographical boundaries for municipalities and parishes cover considerably larger areas than the population centres, such analyses will be less accurate.

The increased risk appears to be due to a general increase in population centres as a whole. There were no indications of specific geographically localized areas involved. Among possible explanations for these findings is a more frequent exposure to viral infections among children in population centres and more densely populated areas. The proportions of young children attending day-care centres in Sweden differ depending on the area of residence. The highest proportion is found in densely populated areas. Viral infections have been shown to be more common in children with regular attendance in day-care centres in Sweden and other countries (Strangert, 1976; Anderson et al, 1988; Petersson and Hakanson, 1989; Harsten et al, 1990). A difference in hospital admission in urban and rural areas for respiratory tract infections in children has been shown in Denmark (Moltesen and Hjuler, 1991).

Our results add to the circumstantial evidence indicating some aetiologically relevant environmental risk factor in population centres and more densely populated areas. The hypothesis of an infectious agent is at present the most plausible among the theories concerning possible environmental risk factors for acute childhood leukaemia.

### REFERENCES

Alexander FE (1993) Viruses, clusters and clustering of childhood leukaemia: a new perspective? *Eur J Cancer* **29A**: 1424–1443

Alexander FE (1995) The search for causes of the leukaemias: comment. *Eur J Cancer* **31A**: 863–867

Anderson LJ, Parker RA, Strikas RA, Farrar JA, Gangarosa EJ, Keyserling HL and Sikes RK (1988) Day-care center attendance and hospitalization for lower respiratory tract illness. *Pediatrics* **82**: 300–308

Aubertin CL and Grellety BP (1923) Contribution à l'etude de la leucemie aigue. *Arch Mal Coeur* **16**: 696–713

Bithell JF (1990) An application of density estimation to geographical epidemiology. *Stat Med* **9**: 691–701

Draper GJ (1991) The geographical epidemiology of childhood leukaemia and non-Hodgkin lymphomas. In *Medical and Population Subjects*, no. 53, pp. 1966–1983. HMSO: London

Greaves MF (1986) Speculations on the cause of childhood acute lymphoblastic leukaemia. *Leukemia* **2**: 120–125

Greaves MF and Alexander FE (1993) An infectious etiology for common acute lymphoblastic leukemia in childhood? *Leukemia* **7**: 349–360

Gustafsson G and Wranne L (1993) 25th anniversary of the Swedish Pediatric Leukemia Group. Quite a number of children with acute lymphatic leukemia are cured. *Lakartidningen* **90**: 2883–2886

Harsten G, Prellner K, Heldrup J, Kalm O and Kornfalt R (1990) Acute respiratory tract infections in children. A three-year follow-up from birth. *Acta Paediatr Scand* **79**: 402–409

Hjalmars U, Kulldorff M and Gustafsson G (1996) Childhood leukemia in Sweden: using GIS and a spatial scan statistic for cluster detection. *Stat Med* **15**: 707–715

Kahn HA and Sempos CT (1989) In *Statistical Methods in Epidemiology*. Oxford University Press: New York

Kinlen L (1995) Epidemiological evidence for an infective basis in childhood leukaemia. *Br J Cancer* **71**: 1–5

Kinlen LJ (1997) High-contact paternal occupations, infection and childhood leukaemia: five studies of unusual population-mixing of adults. *Br J Cancer* **76**: 1539–1545

 © Cancer Research Campaign 1999

Kinlen LJ, Clarke K and Hudson C (1990) Evidence from population mixing in British New Towns 1946–85 of an infective basis for childhood leukaemia. *Lancet* **336**: 577–582

Langford IH and Bentam G (1993) Epidemiology of childhood leukaemia. *Br Med J* **307**: 445–446

McWhirter WR and Bacon JE (1980) Epidemiology of acute lymphoblastic leukaemia of childhood in Brisbane. *Med J Aust* **2**: 154–155

Moltesen B and Hjuler IM (1991) Hospitalization of children in cities and rural areas. 1. Comparative analysis between hospital admission rate and living conditions based on registered data. *Ugeskr Laeger* **153**: 3077–3080

Muirhead CR (1995) Childhood leukaemia in metropolitan regions in the United States a possible relation to population density? *Cancer Causes Control* **6**: 383–388

Parkin DM, Stiller CA, Draper GJ, Bieber CA, Terracini B and Young LJ (1988) *International Incidence of Childhood Cancer*. Publication no. 87. IARC: Lyon

Petersson C and Hakansson A (1989) A prospective study of infectious morbidity and antibiotic consumption among children in different forms of municipal day-care. *Scand J Infect Dis* **21**: 449–457

Ries LAG, Miller BA, Hankey BF, Kosary CL and Harras A (1994) In *SEER Cancer Statistics Review, 1973–1991: Tables and Graphs*. National Cancer Institute, no. 94, p 2789. NIH: Bethesda, MD

Ross JA, Davies SM, Potter JD and Robinson LL (1994) Epidemiology of childhood leukaemia, with a focus on infants. *Epidemiol Rev* **16**: 243–272

Stiller CA and Draper GJ (1998) The epidemiology of cancer in children. In *Cancer in Children*. Voûte PA, Kalifa C and Barret A (eds), pp. 1–20. Oxford University Press: Oxford

Strangert K (1976) Respiratory illness in preschool children with different forms of day care. *Pediatrics* **57**: 191–196

Waller LA, Turnbull BW, Gustafsson G, Hjalmars U and Andersson B (1995) Detection and assessment of clusters of disease: an application to nuclear power plant facilities and childhood leukaemia in Sweden. *Stat Med* **14**: 3–16

Weinstein HJ (1978) Congenital leukaemia and the neonatal myeloproliferative disorders associated with Down’s syndrome. *Clin Haematol* **7**: 147–154

*© Cancer Research Campaign 1999*
